# TROP-2 and Nectin-4 Expression in Muscle-Invasive Urothelial and Rare Non-Urothelial Bladder Carcinoma: Association with Tumour Stage and Clinical Outcome

**DOI:** 10.3390/cancers18162581

**Published:** 2026-08-11

**Authors:** Mohammed Rafea Kanaan, Pouriya Faraj Tabrizi, Jessica Schmitz, Jan H. Bräsen, Markus A. Kuczyk, Hossein Tezval

**Affiliations:** 1 Department of Urology and Urological Oncology, Hannover Medical School, Carl-Neuberg-Strasse 1, 30625 Hannover, Germany; kanaan.mohammed@mh-hannover.de (M.R.K.); farajtabrizi.pouriya@mh-hannover.de (P.F.T.); kuczyk.markus@mh-hannover.de (M.A.K.); 2 Institute of Pathology, Hannover Medical School, Carl-Neuberg-Strasse 1, 30625 Hannover, Germany; schmitz.jessica@mh-hannover.de (J.S.); braesen.jan@mh-hannover.de (J.H.B.)

**Keywords:** bladder cancer, non-urothelial carcinoma, TROP-2, Nectin-4, antibody–drug conjugates, enfortumab vedotin, sacituzumab govitecan, immunohistochemistry

## Abstract

***Background/Objectives:*** Antibody–drug conjugates (ADCs) targeting Nectin-4 (enfortumab vedotin) and TROP-2 (sacituzumab govitecan) have transformed the management of advanced urothelial carcinoma (UC), but evidence on their molecular targets in rare non-urothelial bladder carcinomas (N-UC) is scarce. We evaluated the immunohistochemical expression of TROP-2 and Nectin-4 in muscle-invasive UC and N-UC and assessed their association with tumour stage, nodal status and overall survival. ***Methods:*** In this retrospective single-centre study, 111 consecutive patients with primary muscle-invasive bladder carcinoma (73 UC, 38 N-UC including squamous, adenocarcinoma, neuroendocrine and sarcomatoid variants) were analysed. TROP-2 and Nectin-4 expression was quantified using the H-score; positivity was defined as ≥15. Marker expression was correlated with clinicopathological characteristics and overall survival (OS) using chi-squared, linear-by-linear, log-rank, and Cox regression analyses. ***Results:*** TROP-2 positivity was observed in 46.6% of UC and 36.8% of N-UC (*p* = 0.925); Nectin-4 positivity in 17.8% and 28.9%, respectively (*p* = 0.275). Among positive cases, TROP-2 H-scores were higher in N-UC than in UC (mean 109 vs. 70; Mann–Whitney *p* = 0.045; Cliff’s delta 0.37, 95% CI 0.05–0.66); as no adjustment for multiple testing was applied, this difference is regarded as nominally significant and exploratory. Nectin-4 H-scores were numerically higher in UC (mean 82 vs. 53) but did not reach statistical significance (*p* = 0.84). Across the cohort, TROP-2 expression increased with advancing T stage (linear-by-linear *p* = 0.026) and was associated with nodal involvement (*p* = 0.043). Nectin-4 showed no significant stage association. Sarcomatoid carcinomas were negative for both markers. Median OS was 41 months in UC versus 19 months in N-UC (*p* = 0.668). Neither marker independently predicted OS, whereas advanced T stage (*p* = 0.003) and nodal involvement (*p* = 0.021) were significantly associated with poorer survival; T stage remained independently prognostic in multivariable analysis. ***Conclusions:*** TROP-2 and Nectin-4 are expressed at comparable rates in muscle-invasive UC and rare N-UC, except in sarcomatoid variants. TROP-2 expression increased with advancing tumour stage, suggesting stage-dependent regulation in muscle-invasive disease. Therefore, both markers should be interpreted as potential therapeutic targets whose expression can be demonstrated in these tumours, rather than as prognostic biomarkers or as validated predictive biomarkers of ADC response; because no patient received an ADC, the present study cannot determine whether expression predicts clinical benefit, and this distinction requires prospective, treatment-linked evaluation that includes patients with N-UC.

## Simple Summary

Bladder cancer is one of the most common cancers worldwide. Most bladder cancers are urothelial carcinomas, but a small group of patients has rare non-urothelial types, including squamous, adenocarcinoma, neuroendocrine and sarcomatoid forms. These rare tumours usually present at advanced stages and have worse outcomes, yet they are typically excluded from major clinical trials of new drugs. Two recent targeted therapies for bladder cancer act by binding to surface proteins called TROP-2 and Nectin-4 on tumour cells. Whether these proteins are also present in rare bladder cancer types has not been carefully studied. In this study, we examined tumour tissue from 111 patients with muscle-invasive bladder cancer to compare the expression of TROP-2 and Nectin-4 in urothelial and rare non-urothelial forms. Our findings may help indicate which patients with rare bladder cancers could benefit from these new targeted therapies.

## 1. Introduction

Bladder cancer is the ninth most diagnosed malignancy worldwide, with an estimated 614,000 new cases and 220,000 deaths in 2022 [1]. Urothelial carcinoma (UC) constitutes approximately 90% of bladder cancers in Western countries and serves as the reference histotype for therapeutic development [2]. The remaining approximately 10% comprises a heterogeneous group of non-urothelial carcinomas (N-UC)—including squamous cell carcinoma, adenocarcinoma, neuroendocrine carcinoma, and sarcomatoid variants—that present at more advanced stages and show stage-adjusted survival inferior to conventional UC [2,3]. Because N-UC patients are largely excluded from prospective trials, their treatment is extrapolated from UC data, representing a persistent unmet need [4,5]. Muscle-invasive bladder carcinoma (MIBC) is defined histologically by tumour invasion into the detrusor muscle (≥pT2) and is managed, with curative intent, by radical cystectomy with pelvic lymph-node dissection, increasingly combined with cisplatin-based perioperative chemotherapy and immune-checkpoint inhibition; N-UC variants are generally treated along the same pathways despite limited supporting evidence [5,6].

Antibody–drug conjugates (ADCs) directed against Nectin-4 (enfortumab vedotin, EV) and trophoblast cell surface antigen 2 (TROP-2; sacituzumab govitecan, SG) have substantially reshaped the treatment of advanced UC [7,8]. EV plus pembrolizumab is now a first-line standard [9], whereas the confirmatory TROPiCS-04 trial of SG did not meet its overall survival endpoint [10], refocusing attention on biomarker-driven patient selection. Beyond the metastatic setting, the neoadjuvant SURE-01/02 trials of SG, alone or with pembrolizumab, achieved bladder preservation in approximately 40% of MIBC patients [11,12]. Yet the evidence underpinning ADC approval derives almost exclusively from conventional urothelial histology, leaving the suitability of these targets in N-UC largely uncharacterised.

TROP-2, encoded by *TACSTD2*, is a transmembrane glycoprotein that transduces intracellular calcium signals and promotes tumour-cell proliferation, invasion and survival via PI3K/AKT and MAPK/ERK signalling and epithelial-to-mesenchymal transition [13,14]; its overexpression is associated with adverse features in breast, lung and oral squamous cell carcinoma [15,16,17]. In MIBC, TROP-2 is broadly expressed across basal, luminal and stroma-rich molecular subtypes but markedly reduced in the neuroendocrine subtype, and EV-resistant cell lines retain SG sensitivity, positioning TROP-2 as a potentially complementary rather than redundant target [18]. In upper tract UC (UTUC), however, high TROP-2 expression has been paradoxically associated with papillary morphology, low pathological stage, and favourable progression-free survival [19,20], arguing against a uniform stage–prognosis association across the urothelial tract.

Nectin-4 (*PVRL4*), a nectin-family cell-adhesion molecule, is highly expressed in conventional UC and is the molecular target of EV [21]. However, membranous Nectin-4 frequently decreases during metastatic progression, being absent in more than one third of metastatic UC samples and correlating with reduced EV efficacy [22], and is further attenuated in basal/squamous, neuroendocrine-like and sarcomatoid molecular subtypes [23,24]; in a spatial analysis of 314 muscle-invasive bladder cancers, higher Nectin-4 was associated with lower tumour stage but not overall survival [24]. A comparison of 169 bladder cancers including morphological variants showed that most sarcomatoid and small-cell carcinomas exhibit low Nectin-4 but preserved TROP-2 or HER2 expression [25], supporting histotype-resolved evaluation of multiple ADC targets in parallel.

Despite this clinical relevance, systematic immunohistochemical data on TROP-2 and Nectin-4 in rare non-urothelial histotypes—and their relationship to tumour stage and survival—remain scarce. Given the negative outcome of TROPiCS-04 in unselected populations [10] and the divergent expression of both targets across molecular and morphological subtypes [18,23,24,25], direct characterisation of these targets in N-UC is a prerequisite for rational, target-driven treatment decisions. We therefore evaluated the immunohistochemical expression of TROP-2 and Nectin-4 in a single-centre cohort of muscle-invasive urothelial and rare non-urothelial bladder carcinomas and examined their association with clinicopathological characteristics including tumour stage, nodal status and overall survival.

## 2. Materials and Methods

### 2.1. Study Design and Patient Cohort

This retrospective single-centre study included 111 consecutive patients with primary muscle-invasive bladder carcinoma (≥cT2 confirmed on transurethral resection of the bladder tumour) who underwent radical cystectomy at Hannover Medical School in the period between 2012 and 2025. Sampling was consecutive and non-selective: all patients meeting the inclusion criteria within this period and with evaluable archival tissue were included, without further selection. Immunohistochemical analysis and pathological staging were performed on the cystectomy specimens. No patient received neoadjuvant chemotherapy or any other systemic or radiation therapy before cystectomy; all analyses were therefore performed on treatment-naïve tumour tissue, excluding treatment-induced modulation of TROP-2 or Nectin-4 expression as a confounding factor. Tumours were classified as urothelial carcinoma (UC; *n* = 73) or non-urothelial carcinoma (N-UC; *n* = 38) according to the 2022 World Health Organisation Classification of Tumours of the Urinary System [26]. Clinical and pathological data were obtained from institutional medical records, including age at diagnosis, sex, tumour stage, nodal status, grading, lymphovascular invasion, resection status and follow-up. Tumour staging was performed according to the eighth edition of the TNM classification [27].

### 2.2. Immunohistochemical Analysis of TROP-2 and Nectin-4

Immunohistochemical staining for Nectin-4 and TROP-2 was performed on cystectomy specimens. Nectin-4 was detected using a rabbit recombinant monoclonal antibody (clone EPR15613-68; Abcam, Cambridge, UK; dilution 1:100) and TROP-2 using a rabbit monoclonal antibody (clone SP295; dilution 1:100); for both antibodies, antigen retrieval was performed using Cell Conditioning Solution 1 (CC1; Ventana Medical Systems, Inc., Tucson, AZ, USA). For both markers, only membranous staining of viable tumour cells was scored; cytoplasmic staining, including granular cytoplasmic positivity, was excluded from the assessment. For both NECTIN-4 and TROP-2, only membranous staining of viable tumour cells was considered. The percentages of tumour cells showing weak (1+), moderate (2+), or strong (3+) membranous staining were estimated, and the H-score was calculated as follows: (1 × percentage of 1+ cells) + (2 × percentage of 2+ cells) + (3 × percentage of 3+ cells), yielding a score ranging from 0 to 300. H-scores were classified as negative (0–14), weak (15–99), moderate (100–199), or strong (200–300), as previously described [22,28]. All immunohistochemical stainings were assessed by a single highly experienced uropathologist with more than 30 years of subspecialty experience, who was blinded to clinical outcomes; therefore, interobserver agreement could not be determined.

### 2.3. Statistical Analysis

Descriptive statistics were used to characterise the study cohort. Continuous variables are presented as medians and interquartile ranges (IQRs); categorical variables are reported as absolute and relative frequencies. Comparisons between groups were performed using the Mann–Whitney U/Kruskal–Wallis test for continuous variables and the chi-squared or linear-by-linear test for categorical variables, as appropriate. For the pre-specified comparison of expression intensity between histological groups (H-score among marker-positive tumours), the Mann–Whitney U test was accompanied by the Cliff’s delta effect size with 95% confidence intervals. Associations of H-score with T stage were additionally examined, treating H-score as a continuous variable (Spearman correlation). Overall survival (OS) was estimated by the Kaplan–Meier method and compared between groups using the log-rank test. Univariable and multivariable Cox proportional-hazards regression analyses were performed to identify factors associated with OS. The proportional-hazards assumption was assessed graphically using log-minus-log survival plots and was satisfied for the covariates included. Survival analyses were restricted to the 91 patients (82.0%) with available follow-up data. All statistical analyses were performed using IBM SPSS Statistics version 30.0 (IBM, Armonk, NY, USA). Two-sided *p*-values < 0.05 were considered statistically significant.

Use of Artificial Intelligence (AI) Tools: No generative artificial intelligence tools were used in the preparation of data, figures, or the analytical components of this study. All scientific content, data interpretation, and conclusions are the sole responsibility of the authors.

## 3. Results

### 3.1. Patient Characteristics

The cohort comprised 111 patients with a median age of 69 years (IQR 62–76) and a male predominance (64.9%). UC represented 65.8% of cases and N-UC 34.2%. Within the N-UC subgroup, squamous cell carcinoma was the most frequent subtype (60.5% of N-UC cases, *n* = 23), followed by adenocarcinoma (21.1%, *n* = 8), neuroendocrine carcinoma (10.5%, *n* = 4) and sarcomatoid carcinoma (7.9%, *n* = 3). Median age at diagnosis was 69 years in UC and 67 years in N-UC (*p* = 0.369). A significant male predominance was observed in UC compared with N-UC (75% vs. 49%, *p* = 0.004). The clinicopathological characteristics of the cohort are summarised in Table 1.

All patients had muscle-invasive disease (≥cT2) confirmed on transurethral resection of the bladder tumour (TUR-B). On final cystectomy pathology, most tumours showed advanced local stage—T2 and T3 each comprised 36.0% of cases and T4 tumours 18.0%—whereas a minority were pathologically downstaged to residual T1 (8.1%) or carcinoma in situ (1.8%). Nodal involvement was observed in 42.3% of cases. High-grade tumours (G3) predominated (68.5%), lymphovascular invasion was present in 55.8%, and complete surgical resection (R0) was achieved in 87.4% of patients. No statistically significant differences in T stage, nodal status, grade or resection status were observed between UC and N-UC subgroups (all *p* > 0.05).

### 3.2. TROP-2 Expression and Clinicopathological Associations

**Figure 1:**
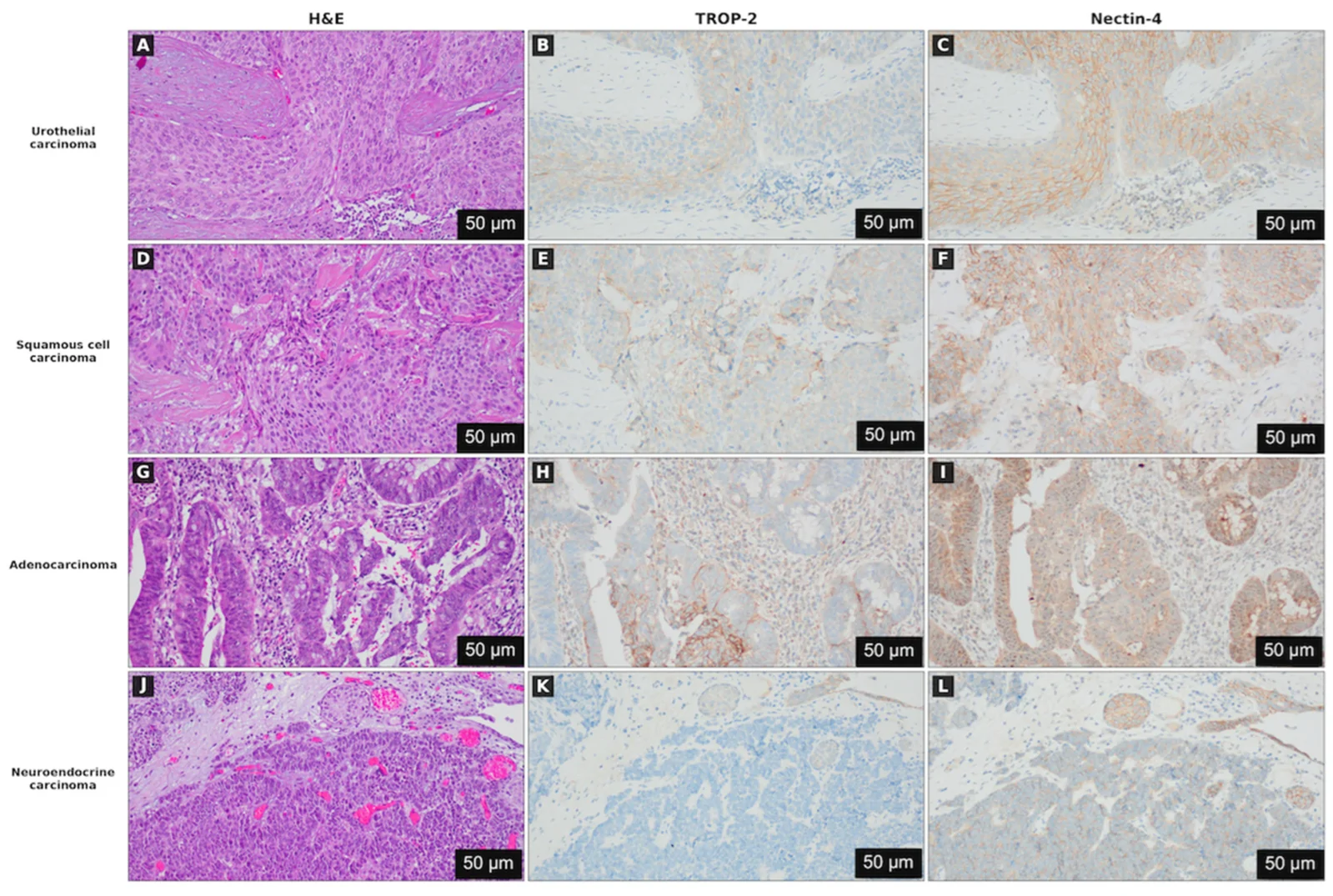
Representative immunohistochemical staining of TROP-2 and NECTIN-4 across histological subtypes of muscle-invasive bladder carcinoma. Composite panels show hematoxylin and eosin staining (left), TROP-2 immunohistochemistry (middle), and NECTIN-4 immunohistochemistry (right) in urothelial carcinoma (**A**–**C**), squamous cell carcinoma (**D**–**F**), adenocarcinoma (**G**–**I**), and neuroendocrine carcinoma (**J**–**L**). Both membranous and cytoplasmic staining patterns were observed, whereas only membranous staining was considered positive and included in the H-score assessment. All images are shown at the same magnification; scale bar, 50 µm.

TROP-2 positivity was detected in 46.6% of UC and 36.8% of N-UC cases, without a statistically significant difference between groups (*p* = 0.925). Within the N-UC subgroup, TROP-2 expression was observed in 39.1% of squamous cell carcinomas, 37.5% of adenocarcinomas, and 50.0% of neuroendocrine carcinomas, whereas sarcomatoid carcinomas showed no detectable expression. Immunohistochemical staining of TROP-2 and Nectin-4 across histological subtypes is shown in Figure 1.

**Figure 2:**
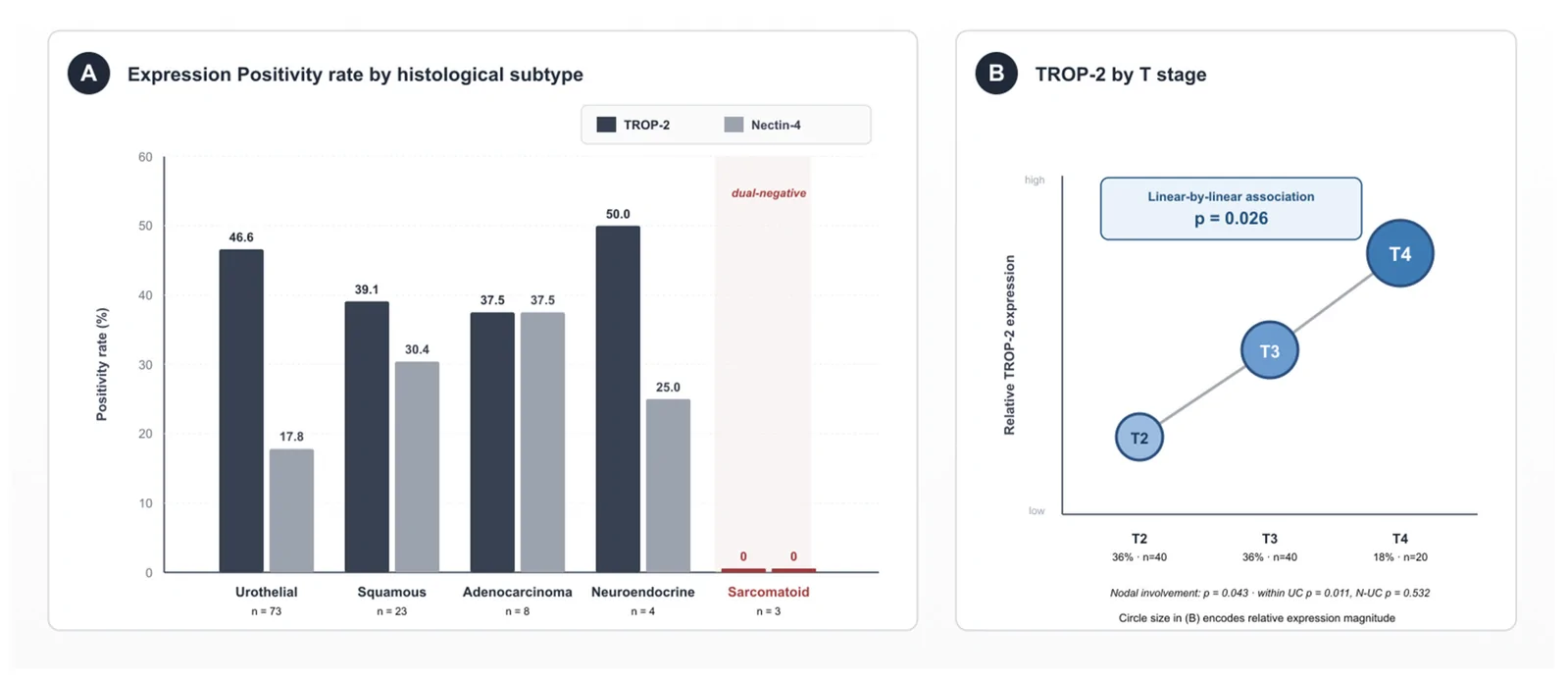
Expression of TROP-2 and Nectin-4 across histological subtypes and tumour stage in muscle-invasive bladder carcinoma (*n* = 111). (**A**) Immunohistochemical positivity rates for TROP-2 and Nectin-4 by histological subtype. (**B**) Stage-dependent TROP-2 expression across pathological T-staging. Circle size encodes relative expression magnitude.

Quantitative assessment revealed substantial inter-subtype variability, with the highest median H-scores observed in neuroendocrine carcinomas (median 60, IQR 0–120), followed by adenocarcinomas (median 25, IQR 5–60), UC (median 10, IQR 1–30) and squamous cell carcinomas (median 5, IQR 1–75). Subtype-specific positivity rates for both markers are summarised in Figure 2A.

Across the entire cohort, TROP-2 expression demonstrated a significant linear increase with advancing T stage (linear-by-linear association *p* = 0.026). The same trend was observed within the UC group (*p* = 0.011) but not in N-UC (*p* = 0.532). When the H-score was analysed as a continuous variable, however, the increase across T2–T4 did not reach statistical significance (Spearman ρ = 0.17, *p* = 0.09), indicating that the stage association is driven mainly by categorical positivity rather than by expression intensity. TROP-2 expression was also significantly associated with nodal involvement (*p* = 0.043), suggesting a link with lymphatic metastatic disease burden (Figure 2B). These stage- and nodal-related associations should be interpreted as exploratory signals rather than established associations.

**Figure 3:**
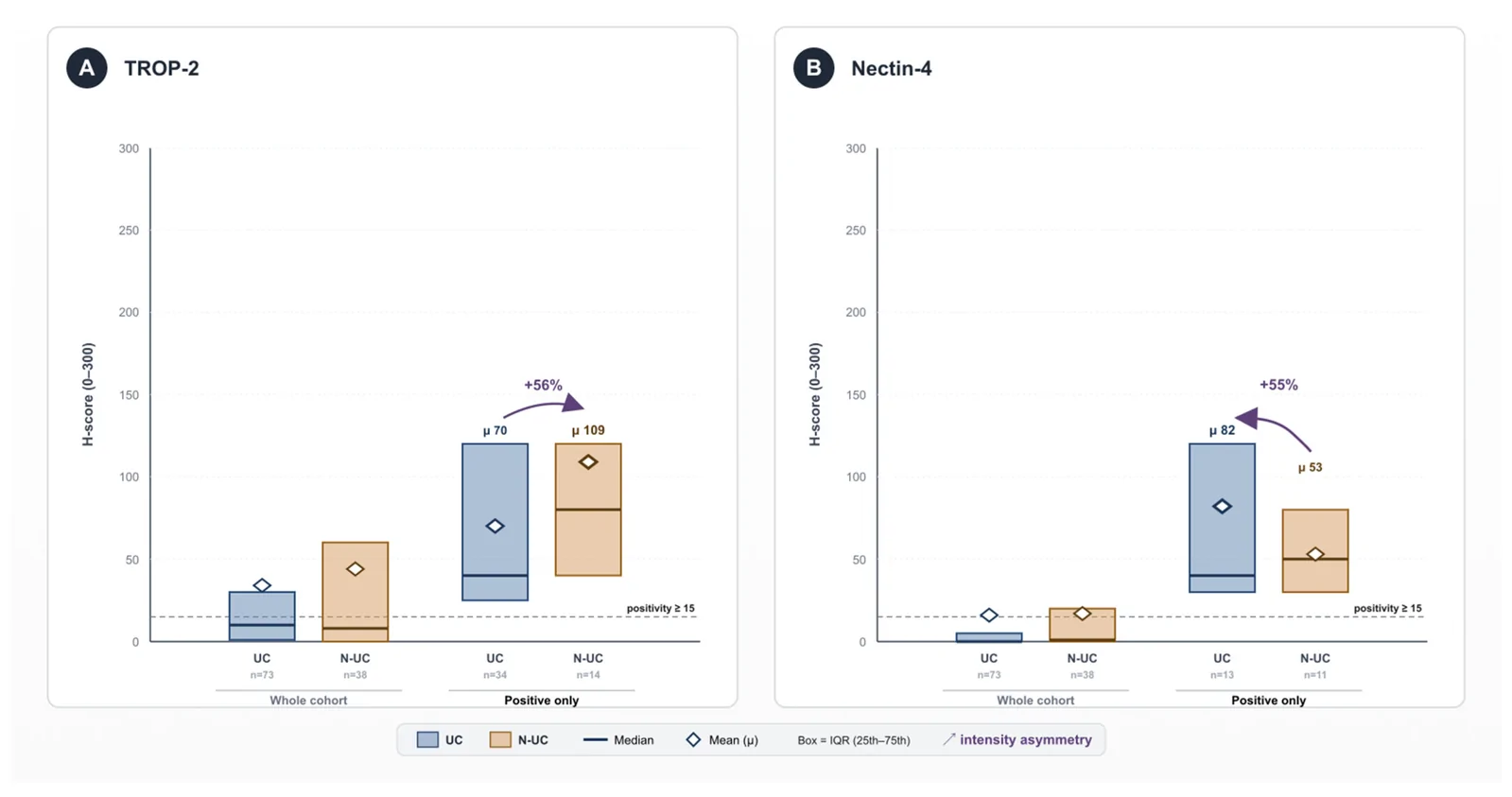
Distribution of TROP-2 (**A**) and Nectin-4 (**B**) immunohistochemical H-scores in urothelial (UC) and non-urothelial (N-UC) bladder carcinoma, stratified by histological group and expression category. Boxes represent the 25–75th percentile (interquartile range), the horizontal line denotes the median, and the diamond marks the mean. Positivity threshold H-score ≥ 15, dashed line.

Notably, although overall TROP-2 positivity rates were comparable between UC and N-UC, the magnitude of expression differed substantially among positive cases. Among TROP-2-positive tumours, the H-score was higher in N-UC than in UC (mean 109, median 80, IQR 40–120 vs. mean 70, median 40, IQR 25–120; *p* = 0.045; Cliff’s delta = 0.37, 95% CI 0.05–0.66), confirming that when N-UC tumours express TROP-2, they do so at a significantly higher intensity than urothelial tumours. Given the modest number of positive cases, these findings warrant confirmation in larger cohorts. Negative tumours (H-score < 15) showed comparably low residual expression in both groups (UC mean 3, median 2; N-UC mean 5, median 1) (Table 2, Figure 3).

### 3.3. Nectin-4 Expression and Clinicopathological Associations

Nectin-4 positivity was observed in 17.8% of UC and 28.9% of N-UC cases (*p* = 0.275). Among the N-UC entities, Nectin-4 expression was detected in 30.4% of squamous cell carcinomas, 37.5% of adenocarcinomas and 25.0% of neuroendocrine carcinomas, whereas no sarcomatoid case showed detectable Nectin-4 expression. Median Nectin-4 H-scores in the whole cohort remained low across all subtypes (UC median 0, IQR 0–5; N-UC median 1, IQR 0–20).

When restricted to positive cases, the relative expression magnitude was inverted compared with TROP-2: among Nectin-4-positive tumours, UC showed higher H-scores than N-UC (mean 82, median 40, IQR 30–120 vs. mean 53, median 50, IQR 30–80); however, this difference was not statistically significant (Mann–Whitney U = 76, *p* = 0.84; Cliff’s delta = 0.06, 95% CI −0.43 to 0.51), with closely overlapping medians, and it should be interpreted with caution given the small number of Nectin-4-positive cases (Table 2, Figure 3). This complementary pattern—higher TROP-2 burden in positive N-UC tumours and higher Nectin-4 burden in positive UC tumours—occurs despite comparable overall positivity rates between groups. A statistically significant association between Nectin-4 expression categories and histological subtype was observed within the N-UC subgroup (*p* = 0.026), whereas no significant association was found within UC (*p* = 0.189). Nectin-4 expression showed no significant association with T stage or nodal status in either subgroup (all *p* > 0.05).

### 3.4. Survival Analysis

**Figure 4:**
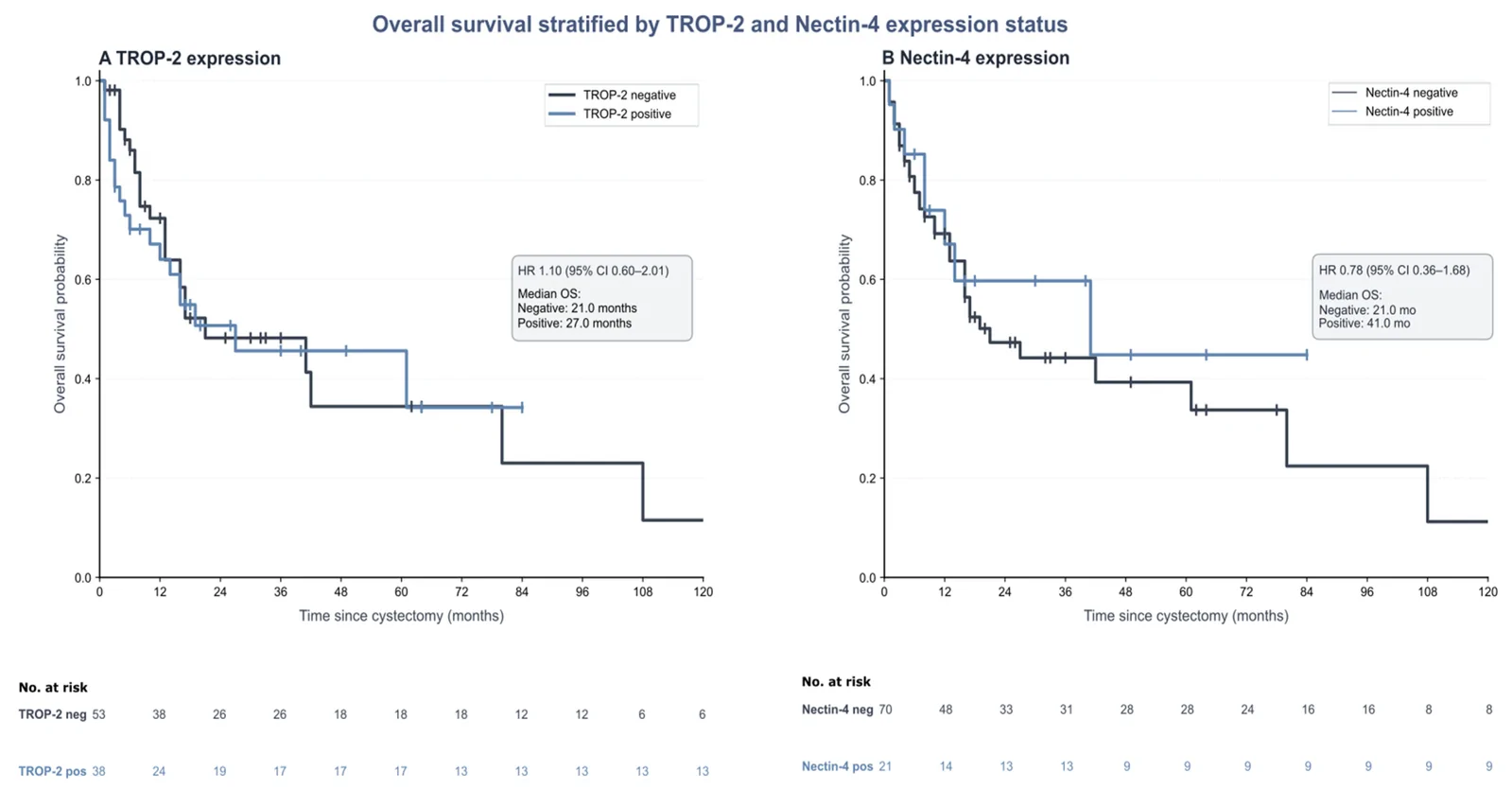
Kaplan–Meier estimates of overall survival stratified by (**A**) TROP-2 expression status and (**B**) Nectin-4 expression status in the overall cohort.

Median overall survival was 41 months in patients with UC and 19 months in those with N-UC, without a statistically significant difference between groups (*p* = 0.668). Kaplan–Meier analysis demonstrated no significant association between TROP-2 expression and OS (log-rank *p* = 0.752; HR 1.10, 95% CI 0.60–2.01, *p* = 0.756). Tumours with high TROP-2 expression showed numerically prolonged median OS compared with low-expression tumours (27 vs. 21 months). Similarly, Nectin-4 expression was not significantly associated with OS (log-rank *p* = 0.515; HR 0.78, 95% CI 0.36–1.68, *p* = 0.522) (Figure 4).

In contrast, advanced pathological T stage (T4 vs. T1; HR 5.81, 95% CI 1.27–26.47, *p* = 0.023) and nodal involvement (N2; HR 2.72, 95% CI 1.32–5.64, *p* = 0.007) were significantly associated with impaired OS in univariable analyses (Table 3).

**Figure 5:**
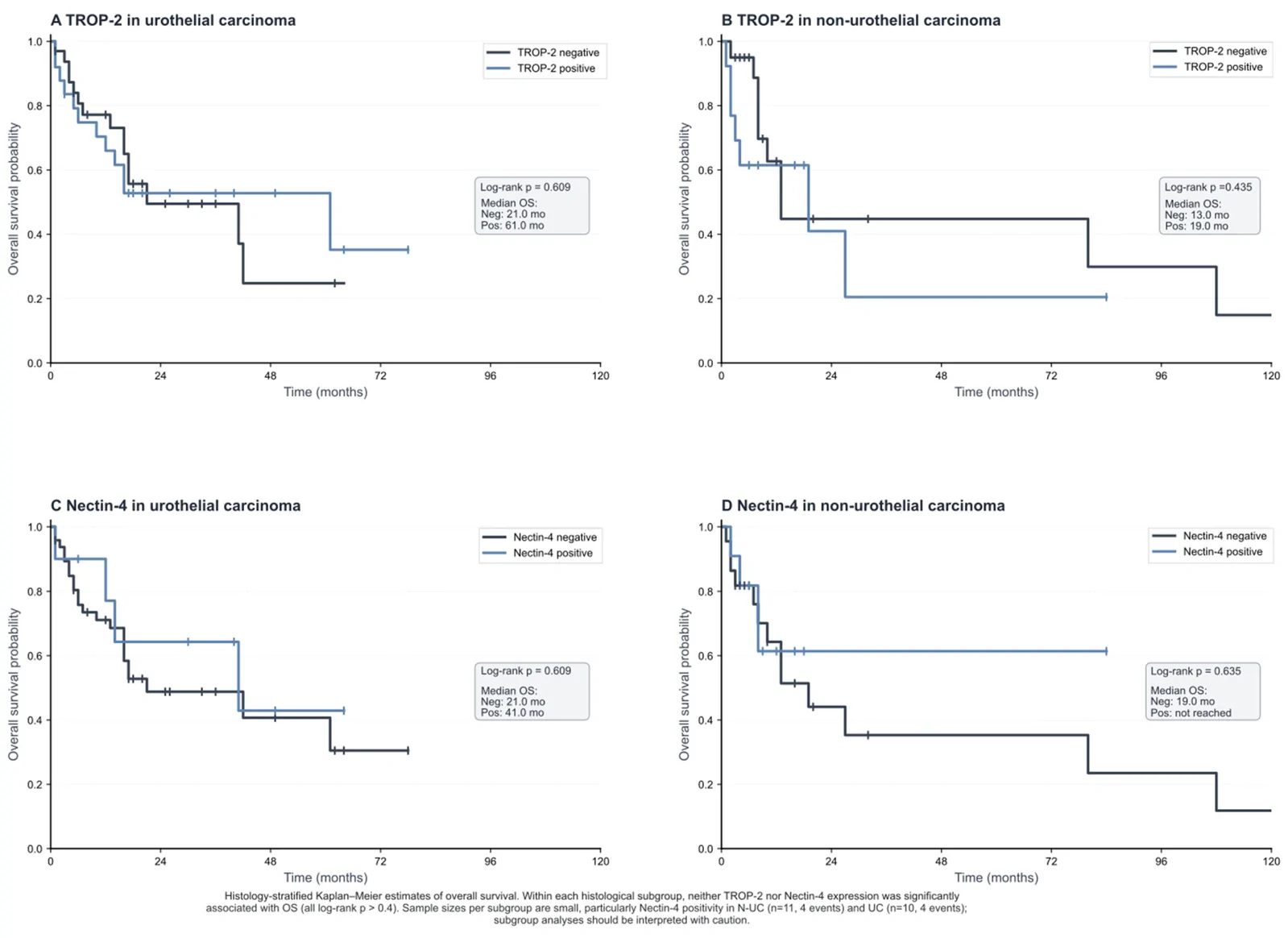
Kaplan–Meier estimates of overall survival stratified by TROP-2 and Nectin-4 expression within the urothelial and non-urothelial subgroups.

Positive resection margin status showed a trend toward worse OS (HR 2.30, 95% CI 0.95–5.56, *p* = 0.064). In multivariable Cox regression including pathological T and N stage, T stage remained independently associated with OS (overall *p* = 0.033), whereas N stage did not retain statistical significance (*p* = 0.335). Within the UC group, tumours with high TROP-2 expression demonstrated numerically prolonged median OS compared with low-expression cases (61 vs. 21 months), although this difference was not statistically significant. Similarly, UC cases with high Nectin-4 expression showed numerically longer OS compared with low-expression cases (41 vs. 21 months). Subgroup analyses stratified by histology (UC vs N-UC) showed no significant association between either marker and OS within either subgroup (all log-rank *p* > 0.05; Figure 5). A separate Cox regression comparing UC versus N-UC alone confirmed that histological grouping did not independently predict survival (HR 0.87, 95% CI 0.46–1.64, *p* = 0.668).

## 4. Discussion

In this single-centre study of 111 patients with muscle-invasive bladder carcinoma, we evaluated the immunohistochemical expression of the ADC targets TROP-2 and Nectin-4 across urothelial and rare non-urothelial histotypes and their association with tumour stage, nodal status and overall survival. Four principal findings emerge: (i) overall positivity rates for TROP-2 (UC 46.6% vs. N-UC 36.8%, *p* = 0.925) and Nectin-4 (UC 17.8% vs. N-UC 28.9%, *p* = 0.275) did not differ significantly between histologies; (ii) among positive tumours, expression intensity differed by histology, with TROP-2 H-scores nominally higher in N-UC than UC (mean 109 vs. 70), whereas Nectin-4 H-scores were higher in UC than N-UC (mean 82 vs. 53); (iii) TROP-2 expression increased significantly with advancing T stage and was associated with nodal involvement, whereas Nectin-4 showed no stage dependence; and (iv) neither marker independently predicted overall survival, whereas T stage and nodal status showed the expected association with survival, with T stage remaining independently prognostic on multivariable analysis.

### 4.1. TROP-2 Expression and Stage-Dependent Regulation

The TROP-2 positivity rate in our urothelial cohort (46.6%) is broadly consistent with previous immunohistochemical series of muscle-invasive bladder cancer, which describe positivity rates between 50% and over 90% depending on the scoring threshold applied [18,23]. In the two-cohort analysis by Bahlinger et al., TROP-2 was widely expressed across luminal, basal/squamous and stroma-rich molecular subtypes but markedly reduced in the neuroendocrine-like subtype, with an overall negativity rate of 6.5% [23]. Chou et al. similarly demonstrated broad TROP-2 expression across most molecular subtypes except neuroendocrine, and EV-resistant cell lines retained SG sensitivity, findings that position TROP-2 as a complementary rather than redundant target to Nectin-4 [18]. Within our N-UC subgroup, TROP-2 detection in neuroendocrine cases contrasts with this published depletion in the molecularly defined neuroendocrine-like subtype, a discrepancy that likely reflects differences between morphologically defined small-cell/neuroendocrine carcinomas and consensus molecular subtype assignment, as well as the small absolute case number (*n* = 4). The absence of TROP-2 in sarcomatoid carcinomas in our series is consistent with prior reports of low or absent expression in sarcomatoid and neuroendocrine variants.

A novel observation in our cohort is the significant linear increase in TROP-2 expression with advancing T stage in urothelial carcinomas (*p* = 0.011) and the overall cohort (*p* = 0.026), together with an association with nodal involvement (*p* = 0.043). However, this stage-dependent upregulation must be interpreted in light of conflicting evidence from the upper urinary tract: both Tomiyama et al. and Kobayashi et al. demonstrated the opposite association in UTUC, with high TROP-2 expression linked to papillary morphology, low pathological stage and favourable progression-free survival [19,29]. At least three non-exclusive explanations are possible: TROP-2 may be biologically regulated differently in the upper versus the lower urinary tract; published UTUC cohorts may be enriched for low-stage and papillary tumours that artificially shift the apparent prognostic direction; or TROP-2 may indeed undergo dynamic upregulation during progression of muscle-invasive bladder cancer, in line with its proposed role in EMT via PI3K/AKT and MAPK/ERK activation [13,14].

This dissociation between target expression and clinical benefit was seen in the neoadjuvant setting: in the SURE-02 trial with SG and pembrolizumab before radical cystectomy, TROP-2 gene expression was associated with neither pathological response (*p* = 0.69) nor recurrence-free survival, whereas molecular subtype and DNA topoisomerase I (TOP1) expression emerged as more promising predictors [12]. Our finding that TROP-2 and Nectin-4 expression levels did not correlate with overall survival is concordant with these observations and reinforces the distinction between a marker that identifies a druggable target and one that quantitatively predicts outcome. Whether the higher TROP-2 intensity we observed in non-urothelial tumours translates into therapeutic benefit therefore cannot be assumed from expression data alone and will require prospective evaluation in histologically diverse cohorts.

### 4.2. Nectin-4 Expression: Subtype Heterogeneity Rather than Uniform Stability

Nectin-4 positivity in our cohort (UC 17.8%, N-UC 28.9%) is at the lower end of the range reported in muscle-invasive bladder cancer, where rates of 30–90% have been described [23,24]. Methodological and biological factors may contribute, including pre-analytical variables, antibody clone, the stringent H-score cut-off of ≥15 applied here, and the high proportion of T3/T4 tumours in our cohort—a stage distribution in which membranous Nectin-4 is reduced relative to lower-stage disease. In a recent spatial analysis of 314 cystectomy specimens by Olah et al., higher Nectin-4 expression was significantly associated with lower tumour stage but not with overall survival [24]. This observation mirrors our own data and reinforces the view that Nectin-4, far from being a static “ubiquitous” antigen, is differentially regulated across stages and subtypes.

Our finding of complete Nectin-4 negativity in sarcomatoid carcinomas is consistent with the published depletion of Nectin-4 in sarcomatoid (17%), squamous (15%), and small-cell/neuroendocrine (0%) histotypes, aligning with the expression pattern of the basal/squamous and mesenchymal-like consensus molecular subtypes [23,24]. Hoffman-Censits et al. showed that sarcomatoid and small-cell carcinomas with low Nectin-4 frequently retain TROP-2 or HER2 expression, a complementarity that supports parallel rather than sequential ADC target assessment in patients with rare histologies [25]. Our observation of preserved TROP-2 expression in squamous (39%), adenocarcinoma (38%), and neuroendocrine (50%) cases, in the context of variable Nectin-4 positivity, illustrates this pattern in a real-world cohort.

The dynamic biology of Nectin-4 acquires clinical importance considering work by Klümper et al., who demonstrated that membranous Nectin-4 expression frequently decreases during metastatic progression and is absent in more than one-third of metastatic UC samples, correlating with reduced EV response [22]. A follow-up study identified NECTIN4 amplifications as predictors of EV response, with response rates exceeding 90% in amplified tumours [30]. These data suggest that Nectin-4 expression at the primary-tumour stage under the setting evaluated here may not fully reflect the target landscape at the time of advanced-disease treatment, and that metastatic biopsy or amplification-based selection may become relevant for future patient stratification.

### 4.3. Asymmetric Expression Intensity in Positive Tumours

A novel and clinically relevant observation in our cohort is that, although TROP-2 and Nectin-4 positivity rates do not differ significantly between UC and N-UC, the magnitude of expression among positive tumours follows an asymmetric pattern. A novel observation in our cohort is that, although TROP-2 and Nectin-4 positivity rates do not differ significantly between UC and N-UC, the magnitude of TROP-2 expression among positive tumours differs by histology. Non-urothelial tumours that express TROP-2 do so with substantially higher mean H-scores than urothelial carcinomas (mean 109 vs. 70), whereas urothelial carcinomas that express Nectin-4 do so at higher intensity than non-urothelial cases (mean 82 vs. 53). This complementarity has been obscured in previous studies that reported only overall medians, which are systematically biased toward zero by the high proportion of negative cases. The pattern parallels at the protein level the morphomolecular observations of Bahlinger et al. and Hoffman-Censits et al., in whose cohorts non-urothelial and variant histotypes with reduced Nectin-4 retained or even enriched TROP-2 or HER2 expression [23,25]. Clinically, this raises the hypothesis that non-urothelial bladder cancers, when TROP-2-positive, may warrant evaluation for TROP-2-directed therapy; the corresponding trend towards higher Nectin-4 intensity in conventional UC did not reach statistical significance and requires confirmation before any clinical inference is drawn. Whether expression magnitude rather than positivity status is a stronger predictor of ADC response deserves prospective evaluation in the ongoing clinical trials [11,12].

### 4.4. Potential Therapeutic Targets Rather than Prognostic Biomarkers

Neither TROP-2 nor Nectin-4 expression was independently associated with overall survival in our cohort, whereas advanced pathological T stage (*p* = 0.003) and nodal involvement (*p* = 0.021) were significantly associated with survival in univariable analysis, with T stage remaining independently prognostic after multivariable adjustment. This dissociation between target expression and outcome is now consistently observed: Bahlinger et al. found no correlation between TROP-2 or Nectin-4 expression and patient survival in either the TCGA-BLCA or the CCC-EMN cohort, and Olah et al. similarly reported no association between Nectin-4 and overall survival despite a clear stage association [23,24]. Taken together, these findings indicate that TROP-2 and Nectin-4 are best understood as therapeutic biomarkers rather than as conventional prognostic biomarkers.

### 4.5. Implications for ADC Therapy in Non-Urothelial Bladder Cancer

The comparable expression of TROP-2 and Nectin-4 in our N-UC and UC cohorts provides a histopathological rationale for considering ADC-based therapy in patients with rare non-urothelial histologies, who are routinely excluded from pivotal trials. It should be emphasised that the considerations set out in this section are hypotheses generated by descriptive expression data; because no patient in this cohort received an ADC, none of them constitutes evidence of therapeutic benefit, and each would require prospective, treatment-linked validation. Emerging real-world evidence is consistent with this rationale, but with important caveats. In the UNITE multi-institutional analysis published by Jindal et al., the presence of histological variants did not preclude EV activity, although squamous-predominant disease was associated with numerically inferior outcomes compared with pure UC [31]. Adequate target expression therefore appears necessary but not sufficient for ADC benefit in non-urothelial histologies; downstream factors—drug internalisation, payload sensitivity, the tumour microenvironment and the molecular subtype of the variant component—appear to co-determine efficacy [23,30]. For patients with pure non-urothelial histology, prospective evidence remains largely absent and inclusion in basket-type ADC trials is urgently needed. Our finding of preserved TROP-2 expression in neuroendocrine and adenocarcinoma cases and preserved Nectin-4 expression in a subset of squamous and adenocarcinoma cases supports the inclusion of these histotypes, with parallel target assessment to identify the most rational ADC choice on a per-patient basis. Conversely, the dual negativity of sarcomatoid carcinomas in our cohort suggests that these patients are unlikely to benefit from currently available ADCs targeting either TROP-2 or Nectin-4 and may instead be candidates for alternative strategies such as HER2-directed approaches [25].

### 4.6. Limitations

Several limitations warrant emphasis. First, the retrospective single-centre design introduces selection bias and limits generalisability. Second, the absolute number of cases per non-urothelial subtype is small, in particular the neuroendocrine (*n* = 4) and sarcomatoid (*n* = 3) groups, so that subtype-level findings are hypothesis-generating rather than subtype-defining and require validation in larger, ideally multicentre, cohorts. Third, all immunohistochemical scoring was performed by a single uropathologist. Although this ensured internal consistency and was carried out by an assessor with more than 30 years of subspecialty expertise, interobserver agreement could not be determined, and a degree of subjective variability cannot be excluded. In addition, the H-score cut-off of ≥15 to define positivity follows prior immunohistochemical studies of TROP-2 and Nectin-4 in urothelial and non-urothelial bladder cancer, in which the same threshold was applied [22,23,24,25], and the identical cut-off was used for both markers here to permit direct comparison. This threshold should be understood as a descriptive positivity cut-off rather than a clinically validated predictive threshold, rather than more recent membranous-only frameworks with superior predictive performance [22]. Fourth, FGFR3 status, PD-L1 expression, and NECTIN4 amplification—all of which have been shown to modulate ADC response [23,30]—were not assessed. Fifth, the primary tumour may not reflect the target landscape of subsequent metastatic disease. Sixth, multiple comparisons were performed across histological subtypes, tumour stage, nodal status and biomarker expression without formal adjustment for multiple testing, so the reported *p*-values are exploratory. Finally, no patient in this cohort received EV or SG, precluding any conclusion regarding the predictive—as opposed to descriptive—value of the observed expression patterns. The markers examined here should accordingly be regarded as potential therapeutic targets whose predictive performance for ADC efficacy remains to be established in prospective, treatment-linked cohorts.

## 5. Conclusions

TROP-2 and Nectin-4 are expressed at comparable frequencies across urothelial and most non-urothelial muscle-invasive bladder carcinomas, except sarcomatoid tumours, which lacked both markers in the few cases examined. Among marker-positive tumours, TROP-2 intensity was significantly higher in non-urothelial than in urothelial carcinoma, whereas the corresponding Nectin-4 difference did not reach significance. Neither marker was associated with survival, and only tumour stage remained independently prognostic. TROP-2 and Nectin-4 represent potential therapeutic targets rather than prognostic biomarkers, and their predictive value for antibody–drug conjugate therapy remains to be established in prospective, treatment-linked studies, particularly in non-urothelial carcinoma. These descriptive findings, particularly those in individual rare subtypes, are exploratory and require prospective, treatment-linked validation in larger, preferably multicentre cohorts before expression-based patient selection can be considered.

## Figures and Tables

**Table 1:** Clinicopathological characteristics of the study cohort (*n* = 111).

Variable	Value
Age, years—median (IQR)	69 (62–76)
Male sex, *n* (%)	72 (64.9)
Urothelial carcinoma, *n* (%)	73 (65.8)
Non-urothelial carcinoma, *n* (%)	38 (34.2)
Squamous cell carcinoma	23 (20.7)
Adenocarcinoma	8 (7.2)
Neuroendocrine carcinoma	4 (3.6)
Sarcomatoid carcinoma	3 (2.7)
Tis, *n* (%)	2 (1.8)
T1, *n* (%)	9 (8.1)
T2, *n* (%)	40 (36.0)
T3, *n* (%)	40 (36.0)
T4, *n* (%)	20 (18.0)
N0, *n* (%)	64 (57.7)
N1, *n* (%)	25 (22.5)
N2, *n* (%)	21 (18.9)
N3, *n* (%)	1 (0.9)
G2/G3, *n* (%)	35 (31.5)/76 (68.5)
Lymphovascular invasion present, *n* (%)	62 (55.8)
R0 resection, *n* (%)	97 (87.4)

IQR, interquartile range.

**Table 2:** TROP-2 and Nectin-4 expression in urothelial versus non-urothelial bladder carcinoma, stratified by expression category.

	Urothelial Carcinoma (*n* = 73)		Non-Urothelial Carcinoma (*n* = 38)	
Variable	Mean	Median (IQR)	Mean	Median (IQR)
**Whole cohort**				
TROP-2 H-score	34	10 (1–30)	44	8 (0–60)
Nectin-4 H-score	16	0 (0–5)	17	1 (0–20)
**Positive cases** **(H-score ≥ 15)**				
TROP-2 H-score	70	40 (25–120)	109	80 (40–120)
Nectin-4 H-score	82	40 (30–120)	53	50 (30–80)
**Negative cases** **(H-score < 15)**				
TROP-2 H-score	3	2 (0–5)	5	1 (0–5)
Nectin-4 H-score	2	0 (0–2)	2	0 (0–1)
**Demographics and positivity**				
Median age, years	—	69	—	67
Male sex, *n* (%)	54 (75.0)		18 (49.0)	
TROP-2 positive, *n* (%)	34 (46.6)		14 (36.8)	
Nectin-4 positive, *n* (%)	13 (17.8)		11 (28.9)	
T2–T3 stage, *n* (%)	56 (76.7)		24 (63.2)	
Node-negative (N0), *n* (%)	42 (57.5)		22 (57.9)	

IQR, interquartile range. Note the histology-dependent expression intensity among positive cases: non-urothelial carcinomas show significantly higher TROP-2 H-scores than urothelial carcinomas (mean 109 vs. 70; nominally significant, exploratory), whereas the higher Nectin-4 H-scores in urothelial cases (mean 82 vs. 53) did not reach statistical significance, despite comparable overall positivity rates.

**Table 3:** Univariable Cox regression analysis for overall survival.

Variable	HR	95% CI	*p*-Value
TROP-2 high expression	1.10	0.60–2.01	0.756
Nectin-4 high expression	0.78	0.36–1.68	0.522
UC vs. N-UC histology	0.87	0.46–1.64	0.668
T stage (T4 vs. T1)	5.81	1.27–26.47	0.023
N stage (N2)	2.72	1.32–5.64	0.007
Positive resection margin	2.30	0.95–5.56	0.064

HR, hazard ratio; CI, confidence interval; UC, urothelial carcinoma; N-UC, non-urothelial carcinoma.

## Data Availability

The de-identified patient-level dataset supporting the findings of this study, including per-case TROP-2 and Nectin-4 H-scores and clinicopathological variables, is available from the corresponding author on reasonable request, subject to institutional data-protection regulations and a data-transfer agreement. Restrictions apply to public deposition because the data derive from clinical records of a rare-tumour cohort in which full anonymisation cannot be guaranteed.

## Abbreviations

ADC	antibody–drug conjugate
CI	confidence interval
EMT	epithelial-to-mesenchymal transition
EV	enfortumab vedotin
HR	hazard ratio
IHC	immunohistochemistry
IQR	interquartile range
MIBC	muscle-invasive bladder carcinoma
N-UC	non-urothelial carcinoma
OS	overall survival
SG	sacituzumab govitecan
TROP-2	trophoblast cell surface antigen 2
UC	urothelial carcinoma
UTUC	upper tract urothelial carcinoma
